# Luteolin in Inflammatory Bowel Disease and Colorectal Cancer: A Disease Continuum Perspective

**DOI:** 10.3390/cimb47020126

**Published:** 2025-02-14

**Authors:** Fang Liu, Cui Guo, Xue Liu, Zhili Gu, Wenxuan Zou, Xuegui Tang, Jianyuan Tang

**Affiliations:** 1 Clinical School of Medicine, Chengdu University of Traditional Chinese Medicine, Chengdu 610072, China; liufang@stu.cdutcm.edu.cn (F.L.); gc__123@163.com (C.G.); 2 Clinical Medicine College of Integrated Chinese and Western Medicine, North Sichuan Medical College, Nanchong 637100, China; lx2526253694@163.com (X.L.); guzhili2207@163.com (Z.G.); zwx23079@163.com (W.Z.)

**Keywords:** luteolin, inflammatory bowel disease, colorectal cancer, ulcerative colitis, natural products

## Abstract

Inflammatory bowel disease (IBD) is a chronic intestinal inflammatory condition that may progress to colorectal cancer (CRC), presenting significant challenges to global health. With shifts in lifestyle, the incidence of both conditions continues to rise, underscoring the urgent need for effective treatments. While traditional therapies can be effective, their high recurrence rates and associated adverse reactions limit their broader application. Luteolin, a flavonoid derived from natural plants, has emerged as a promising focus in both IBD and CRC research due to its multi-target therapeutic potential. This article reviews the molecular mechanisms and signaling pathways through which luteolin regulates immune cell differentiation, mitigates inflammation and oxidative stress, modulates gut microbiota, and restores intestinal mucosal barrier function in IBD. In the context of CRC, luteolin demonstrates significant anti-tumor effects by inhibiting cancer cell proliferation, inducing apoptosis, and suppressing cell migration and invasion. Notably, luteolin has demonstrated significant improvements in IBD symptoms by influencing the differentiation of T cell subsets, decreasing the expression of inflammatory mediators, activating antioxidant pathways, and enhancing the structure of gut microbiota. Furthermore, advancements in formulation technology, such as the use of polymer micelles and responsive nanoparticles, have greatly improved the bioavailability and efficacy of luteolin. However, further investigation is needed to address the bioavailability and potential toxicity of luteolin, particularly in the critical transition from IBD to CRC. This article emphasizes the potential of luteolin in the treatment of IBD and CRC and anticipates its promising prospects for future clinical applications as a natural therapeutic agent.

## 1. Introduction

Inflammatory bowel disease (IBD) is a non-specific intestinal inflammatory disorder with unknown etiology [1]. Its clinical manifestations are varied and include diarrhea, abdominal pain, hematochezia, and weight loss. In severe cases, it may be accompanied by serious complications such as gastrointestinal bleeding, intestinal obstruction, and even malignancy, exhibiting a chronic and recurrent disease course [2]. IBD is primarily categorized into two major types: ulcerative colitis (UC) and Crohn’s Disease (CD) [3]. In UC, lesions are predominantly confined to the colonic mucosa and submucosa, typically beginning in the rectum and potentially extending proximally to involve the entire colon. The hallmark symptom of UC is bloody diarrhea [4]. Conversely, CD lesions are more extensive and can affect any segment of the gastrointestinal tract, characterized by discontinuous transmural inflammation, particularly prevalent in the right colon and terminal ileum. This condition can readily lead to complex complications such as intestinal perforation, fistulas, and abscesses [5].

One of the most serious complications of IBD is colorectal cancer (CRC). Studies have shown that chronic intestinal inflammation in IBD patients significantly increases their risk of developing CRC. The risk of CRC in IBD patients is 2–3 times higher than that in the general population, and the risk increases with the duration and extent of disease. The pathogenesis of IBD-associated CRC involves multiple factors, including chronic inflammation, oxidative stress, and dysregulation of the immune system. Persistent inflammation leads to DNA damage, alterations in cell signaling pathways, and changes in the intestinal microenvironment, all of which contribute to the development of CRC. Furthermore, the progression from IBD to CRC typically follows an inflammation–dysplasia–carcinoma sequence, which differs from the traditional adenoma–carcinoma sequence seen in sporadic CRC [6].

In recent years, the incidence and prevalence of inflammatory bowel disease (IBD) have exhibited a significant upward trend globally. It is estimated that approximately 10 million individuals worldwide are affected by IBD [7]. Despite its widespread impact, the precise cause and pathogenesis of IBD remain unclear, presenting substantial challenges to patients’ daily lives [8]. Currently, the medical community generally recognizes that multiple factors—including genetic susceptibility, environmental influences, immune dysfunction, gut microbiota imbalance, and impaired intestinal mucosal barrier function—contribute to the onset and progression of IBD [9]. Clinically, commonly employed treatments include aminosalicylates, glucocorticoids, immunosuppressants, biological agents, and other pharmaceuticals. However, the issues of high recurrence rates and numerous adverse reactions following drug withdrawal require urgent attention [10]. Consequently, a thorough investigation into the pathogenesis of IBD, the identification of new therapeutic targets, and the development of safer and more effective treatments are of paramount importance for enhancing the quality of life and prognosis of patients with IBD.

**Figure 1:**
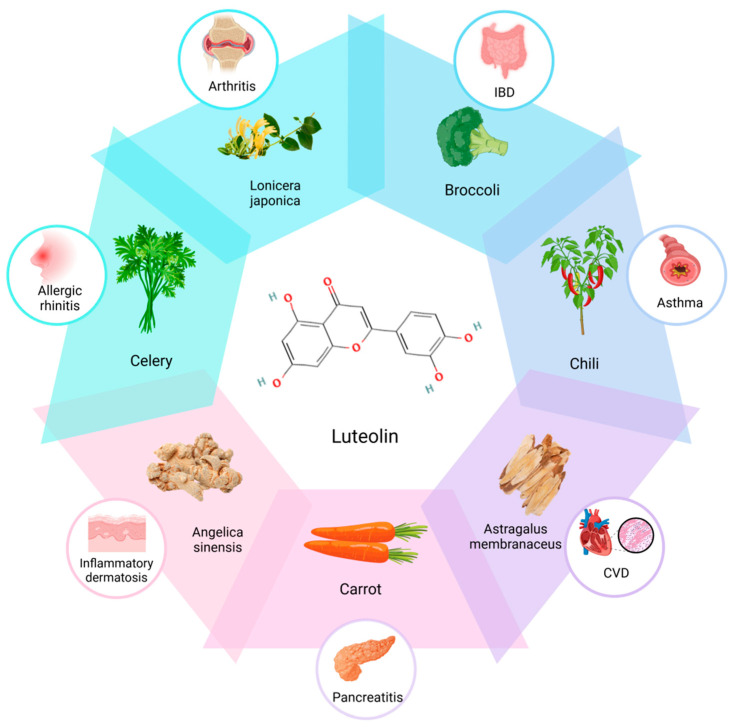
Luteolin for the treatment of various inflammatory diseases and their sources.

Natural products are widely utilized in the prevention and treatment of various chronic diseases due to their multiple targets and high safety profile. In recent years, luteolin has garnered significant attention as a potential treatment for inflammatory bowel disease (IBD) [11]. Luteolin is a flavonoid compound predominantly found in glycoside form and is commonly present in fruits, vegetables, and medicinal herbs (Figure 1), such as honeysuckle, celery, pepper, broccoli, carrot, astragalus, and angelica [12,13,14,15,16]. Research indicates that luteolin acts as a natural antioxidant with potent anti-inflammatory, anti-diabetic, and cardioprotective properties. Numerous scholars have undertaken extensive research on luteolin’s effects in the treatment of IBD from various perspectives, revealing that it can regulate immune cell differentiation, mitigate inflammatory responses and oxidative stress, modulate gut microbiota, and restore the integrity of the intestinal mucosal barrier [17,18,19,20,21]. Luteolin exhibits multiple beneficial effects, demonstrating significant advantages in preventing disease recurrence and effectively controlling the onset and progression of IBD. Consequently, this article aims to provide a comprehensive review of the potential molecular mechanisms and associated signaling pathways through which luteolin may exert its therapeutic effects in IBD, thereby offering valuable insights for the clinical application of natural products in the management of this condition.

## 2. Luteolin Properties

As a member of the flavonoid family, luteolin possesses a range of unique biological and chemical properties, including anti-inflammatory, antioxidant, and anti-cancer effects, which establish its potential application in the treatment of inflammatory bowel disease (IBD) [22,23]. Luteolin manifests as bright yellow, needle-like crystals, exhibiting a slight affinity for water molecules and is only soluble in trace amounts. It displays weak acidic characteristics, with solubility significantly increasing in alkaline environments, while remaining stable under normal conditions, thus facilitating the regulation of its release and absorption in the intestinal environment [24]. Moreover, luteolin is readily soluble in ethanol and ether, but shows limited solubility in hot water and is nearly insoluble in cold water. Its aqueous solution exhibits an elegant yellow hue, which deepens to dark yellow when mixed with a 10% sodium hydroxide solution [25]. Additionally, when reacted with concentrated sulfuric acid, a zinc salt is produced, resulting in a dark red and yellow solution. These color changes serve as a strong basis for the identification of luteolin [26].

Luteolin exhibits significant solvent-dependent redox behavior in the field of electrochemistry, particularly highlighted by the notable differences in redox peak shapes observed in dimethylformamide (DMF) and ethyl cyanide [27]. This finding underscores the influence of the solvent environment on its electrochemical properties. Additionally, luteolin demonstrates a significant regulatory effect. The phenolic hydroxyl group present in the luteolin molecule imparts a weak acidic character to its aqueous solution and facilitates complex reactions with various metal ions, leading to the formation of colorful complexes. This property renders luteolin useful in analytical chemistry [28]. Furthermore, in the field of medicine, luteolin has garnered considerable attention due to its extensive range of biological activities, especially its potential application in the treatment of chronic inflammation and other gastrointestinal diseases [29].

## 3. Methodology

To explore the therapeutic effects of luteolin on inflammatory bowel disease and colorectal cancer, we conducted a systematic literature review following PRISMA guidelines. Our search strategy encompassed four major databases: PubMed, Web of Science, Embase and China’s National Knowledge Infrastructure, covering publications from database inception to November 2024. The search terms included combinations of “luteolin”, “3′,4′,5,7-tetrahydroxyflavone” with terms related to the target conditions (“Inflammatory bowel disease”, “IBD”, “Ulcerative colitis”, “Crohn’s disease”, “Colorectal cancer”, “CRC”).

Our initial search identified 1636 potentially relevant articles (PubMed *n* = 583, Web of Science *n* = 512, Embase *n* = 421, CNKI *n* = 120). Studies were first screened based on titles and abstracts using the following inclusion criteria: (1) Original research articles and systematic reviews published in peer-reviewed journals; (2) studies investigating luteolin’s mechanisms in IBD or CRC; (3) in vitro, in vivo, or clinical studies with clear methodology; (4) full-text articles available in English or Chinese. We excluded conference abstracts, case reports, letters to editors, studies focusing solely on extraction methods, and articles without experimental validation.

**Figure 2:**
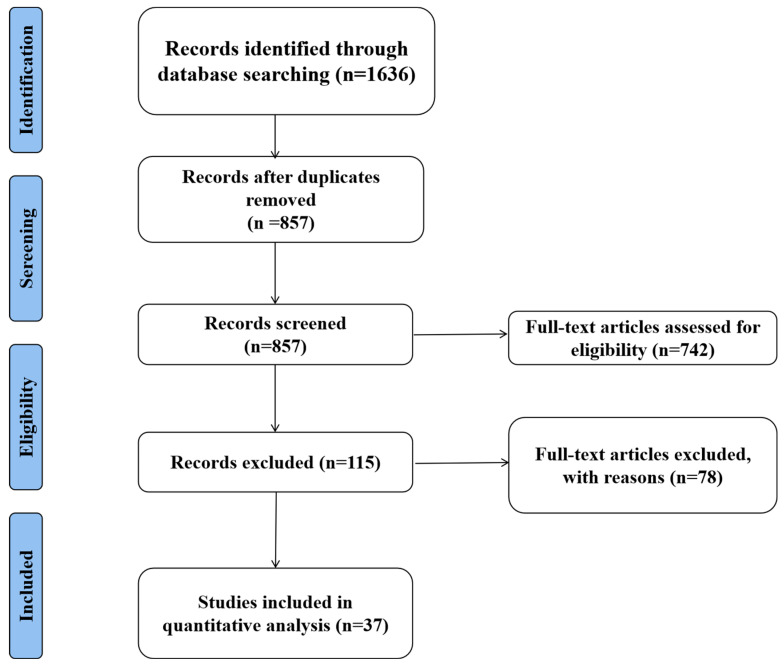
Flowchart of the literature search and study selection.

After removing duplicates (*n* = 425) and screening titles/abstracts (*n* = 1211), 156 full-text articles were assessed for eligibility. The final analysis included 37 articles that met all criteria (Figure 2).

## 4. Multi-Pathway Application of Luteolin in Inflammatory Bowel Disease (IBD)

### 4.1. Regulation of Immune Cell Differentiation

Inflammatory bowel disease is a chronic inflammatory disease involving abnormal responses of intestinal immune cells. Especially in ulcerative colitis, the pathological process involves the activation and differentiation of a variety of immune cells, mainly including T cells and macrophages [30]. Luteolin has been shown to have a positive effect on regulating immune cell differentiation [31].

At the T cell level, luteolin alleviates immune imbalance in inflammatory bowel disease (IBD) by influencing the differentiation of T cell subsets. It particularly promotes the generation of regulatory T cells (Treg) while inhibiting the differentiation of inflammatory Th1 and Th17 cells. Recent research has developed reactive oxygen species (ROS)-responsive nanoparticles (NPs) to facilitate the targeted delivery of luteolin to the inflamed colon via oral administration, thereby enhancing its therapeutic effect. This nanomedicine effectively modulates the inflammatory microenvironment by regulating the balance between Th1/Th2 and Th17/Treg cells [32]. Specifically, the combination of luteolin and nanotechnology reduces inflammation and accelerates intestinal mucosal healing by increasing the number of Treg and Th2 cells while decreasing the number of Th1 and Th17 cells.

Moreover, the regulation of macrophages by luteolin cannot be ignored. In a DSS-induced mouse colitis model, luteolin significantly improved colitis symptoms by inhibiting macrophage activation and chemotaxis. Specific mechanisms include the inhibition of phosphorylation of IKK α/β and subsequently blocking the activation of NF-kB signaling pathway, a key pathway controlling the inflammatory response in macrophages [33,34]. Subsequently, it reduces the levels of inflammatory factors, including tumor necrosis factor alpha (TNF-α), interleukin-1 (IL-1), and interleukin-6 (IL-6), playing a role in alleviating inflammation. Studies suggest that luteolin also reduces the migratory behavior of macrophages, which may contribute to reduce immune cell infiltration at sites of inflammation.

### 4.2. Reduction in the Inflammatory Response and Reactive Oxygen Species (ROS)

IBD is a chronic intestinal inflammatory disease involving complex immune responses and oxidative stress processes. Ulcerative colitis (UC), in particular, is characterized by persistent inflammation of the intestinal mucosa, associated with the overproduction of reactive oxygen species (ROS) and weakened antioxidant defenses [35]. Luteolin has shown potential therapeutic effects on IBD in multiple studies.

In a dextran sulfate sodium (DSS)-induced mouse colitis model, luteolin significantly reduced the disease activity index (DAI), colon shortening, and histological damage. It effectively mitigates the inflammatory response by decreasing the expression of inflammatory mediators such as inducible nitric oxide synthase (iNOS), tumor necrosis factor (TNF-α), and interleukin-6 (IL-6). Additionally, luteolin lowered colonic dialdehyde (MDA) levels, a biomarker of lipid peroxidation, indicating its antioxidant effects [36]. The antioxidant properties of luteolin are associated with its activation of the nuclear factor erythroid 2-related factor 2 (Nrf2) signaling pathway. This pathway activation enhances the expression of antioxidant enzymes, including heme oxygenase-1 (HO-1) and quinone oxidoreductase 1 (NQO-1), which are crucial for maintaining intracellular redox balance [37]. Luteolin treatment also increased the activities of colonic superoxide dismutase (SOD) and catalase (CAT), key components of the cellular antioxidant defense system that help mitigate oxidative stress damage [38]. Furthermore, a separate study employing liquid chromatography–mass spectrometry conducted a metabolomic analysis of the serum from UC rats treated with luteolin. This analysis revealed that luteolin can reduce colon damage and inflammation, enhance serum antioxidant properties, and lower propylene glycol aldehyde levels, further demonstrating its robust antioxidant properties [11].

In addition, related studies have found that the regulation effect of luteolin on serotonin (5-HT) levels is also related to its anti-inflammatory mechanism. 5-HT is an important neurotransmitter, and its overproduction is implicated in the pathogenesis of IBD. Luteolin reduced the generation of 5-HT by inhibiting TPH-1 expression and the MAPK/ERK signaling pathway, thus producing an inhibitory effect on intestinal inflammation [39].

### 4.3. Regulate Gut Microbiota

The intestinal microbiota, commonly referred to as the gut microbiota, comprises trillions of microorganisms that participate in various physiological processes, including food digestion, energy metabolism, immune system development, and pathogen defense. In patients with inflammatory bowel disease (IBD), the composition of the gut microbiota undergoes significant alterations, a phenomenon known as dysbiosis [40,41], which may exacerbate the pathological processes of IBD by producing inflammatory mediators and compromising intestinal barrier function. Research indicates that luteolin can influence the diversity and composition of the intestinal microbiota, potentially benefiting the treatment of IBD. For instance, 16S rDNA sequencing analysis revealed that luteolin alters the composition of the gut microbiota in ulcerative colitis (UC) rats, with *Lactobacillus*, *Bacteroides*, *Roseburia*, and *Butyricococcus* emerging as the dominant genera in the luteolin-treated group [42]. Another study found that intraperitoneal (IP) injection of luteolin increased the abundance of beneficial bacteria such as *Akkermansia muciniphila*. These microorganisms are capable of producing short-chain fatty acids (SCFAs), which play a crucial role in maintaining intestinal barrier function and exerting anti-inflammatory effects [43].

Moreover, the regulatory effect of luteolin on intestinal microflora may be closely related to its anti-inflammatory effect. The was found that luteolin treatment could significantly reduce colon injury and inhibit colon inflammation in the UC (ulcerative colitis) rat model, which was associated with the decrease in NF-kB, IL-17, and IL-23 and the increase in PPAR-γ [42]. Luteolin treatment was able to significantly reduce the levels of pro-inflammatory cytokines such as IL-6 and IL-1 β in the UC rat model, and then narrowed the range of inflammatory environment.

### 4.4. Repair of the Intestinal Mucosal Barrier Function

**Figure 3:**
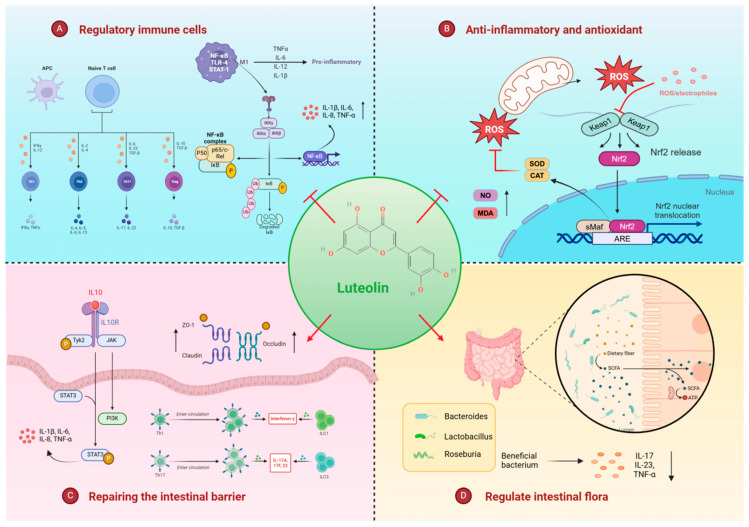
Luteolin multi-pathway treatment of IBD. (Luteolin exerts its therapeutic effects through four main pathways: (**A**) Regulation of immune cell differentiation—promoting Treg cell generation while inhibiting inflammatory Th1/Th17 cells and macrophage activation; (**B**) reduction in inflammatory response and ROS—decreasing inflammatory mediators (iNOS, TNF-α, IL-6) and activating antioxidant pathways (Nrf2/HO-1); (**C**) repair of intestinal mucosal barrier—enhancing tight junction proteins (ZO-1, Occludin) expression and promoting intestinal epithelial restoration through the SHP-1/STAT3 pathway); (**D**) regulation of gut microbiota—increasing beneficial bacteria (*Lactobacillus*, *Bacteroides*) and modulating microbiota composition.

UC is a chronic inflammatory bowel disease characterized by damage to the intestinal mucosal barrier, which not only exacerbates the inflammatory process but also facilitates the penetration of inflammatory mediators [43]. The integrity of the intestinal mucosal barrier is crucial for maintaining intestinal homeostasis. Studies have demonstrated that luteolin promotes the repair of the damaged intestinal barrier by regulating the balance of group 3 innate lymphocyte (ILC3) subsets. In a dextran sulfate sodium (DSS)-induced UC mouse model, luteolin significantly alleviated UC symptoms, including the prevention of weight loss and the reduction in the disease activity index (DAI), while enhancing the expression of intestinal barrier function-related proteins ZO-1 and Occludin [43]. Further mechanistic studies indicated that luteolin may exert its effects through the SHP-1/STAT3 signaling pathway, which is essential for preserving the integrity of the intestinal epithelial barrier [44]. The luteolin treatment increased the expression of tight junction proteins and decreased the levels of pro-inflammatory cytokines, such as IL-17a and INF-γ, while promoting the production of the anti-inflammatory cytokine IL-22. These changes are linked to the promoting effect of luteolin on the conversion of NCR+ILC3 to NCR-ILC3 and are associated with the secretion of IL-22 via the Notch pathway [43]. Collectively, these studies suggest that luteolin may represent a promising therapeutic approach for the treatment of UC, with its effects possibly related to several mechanisms of appeal (Figure 3).

### 4.5. Application of Various Luteolin Preparations in Inflammatory Bowel Disease (IBD)

As a natural compound with anti-inflammatory and antioxidant properties, its potential in the treatment of inflammatory bowel disease (IBD) has been widely recognized by the scientific community. Recent studies on drug preparation technology provide a new perspective on the application of luteolin. An innovative approach is by using polymeric micelles as a drug carrier. Luteolin-coated polymer micelles were prepared from a synthetic RA-SS-mPEG polymer material [45]. This novel formulation showed significant anti-inflammatory effects in animal models, with significantly improved bioavailability and extended half-life. This suggests that the polymeric micellar technique is able to significantly improve the efficacy of luteolin, while potentially reducing the required dose and side effects.

Furthermore, responsive nanoparticle technology offers new opportunities for the targeted delivery of luteolin. The reactive oxygen species (ROS)-responsive nanoparticles developed by the researchers can release luteolin in response to inflammation, thereby enhancing the local concentration and efficacy of the drug. These nanoparticles demonstrated significant therapeutic effects in animal models of colitis, effectively alleviating inflammatory symptoms and modulating the inflammatory microenvironment [32]. Another noteworthy study involves the nanocomplex formed by the coordination of cerium ions and luteolin. This cerium–luteolin nanocomplex (CeLutNCs) not only exhibits excellent antioxidant capacity but also has the ability to modulate immune responses, indicating therapeutic potential in various inflammation-related diseases [46]. Animal experiments revealed that CeLutNCs significantly improved kidney function and alleviated lung damage, highlighting their potential in treating IBD.

## 5. Multi-Pathway Application of Luteolin in Colorectal Cancer (CRC)

### 5.1. Inhibition of Colorectal Cancer Cell Proliferation and Induction of Apoptosis

In the development of colorectal cancer (CRC), abnormal cell proliferation and resistance to apoptosis are key pathogenic factors. Cancer cells overcome normal cell cycle control and continue to proliferate, leading to tumor formation [47]. Luteolin significantly inhibits cancer cell proliferation by regulating the cell cycle and promoting apoptosis in CRC cells [48,49]. Research has shown that luteolin downregulates the expression of Cyclin D1 and CDK4, blocking the transition of CRC cells from the G1 phase to the S phase, thereby reducing cell proliferation [50]. In addition, luteolin activates Caspase-3 and Caspase-9, triggering the mitochondrial pathway to promote cell death. By modulating the Bax/Bcl-2 ratio, luteolin enhances apoptosis in CRC cells, eliminating abnormally proliferating tumor cells [51].

Luteolin also enhances the apoptotic response by regulating the PI3K/Akt pathway, which plays a critical role in the growth, proliferation, and survival of cancer cells. Studies indicate that luteolin inhibits the activation of the PI3K/Akt signaling pathway, reducing the phosphorylation of Akt and mTOR, thus inhibiting CRC cell survival and enhancing the apoptotic effect [48]. These findings suggest that luteolin not only directly suppresses CRC cell proliferation to slow tumor growth, but also promotes apoptosis to eliminate cancer cells with abnormal growth [52].

### 5.2. Inhibition of Colorectal Cancer Cell Migration and Invasion

Colorectal cancer (CRC) mortality is predominantly attributed to its metastatic capabilities. Cancer progression is characterized by CRC cells’ ability to migrate and invade, with the epithelial–mesenchymal transition (EMT) playing an essential role in tumor cell metastasis [53]. Studies have revealed that luteolin possesses potent inhibitory effects against CRC metastasis. Specifically, through its suppressive effect on matrix metalloproteinases (MMP-2, MMP-9), luteolin impairs the ability of cancer cells to degrade the extracellular matrix (ECM), thus hindering CRC cell invasion [54]. Additionally, luteolin’s regulatory effect on EMT involves the enhancement of E-cadherin levels while simultaneously reducing N-cadherin and Vimentin expression, resulting in decreased cancer cell mobility and metastatic potential [55].

The underlying mechanism involves luteolin’s interference with the Wnt/β-catenin pathway signaling cascade. By preventing the translocation of β-catenin to the nucleus, luteolin effectively blocks the transcription of genes responsible for CRC cell proliferation, migration, and metastatic behavior [56,57]. Through these multiple molecular targets, luteolin exhibits comprehensive anti-metastatic properties, affecting both the invasive capacity and phenotypic characteristics of cancer cells, thereby impeding the metastatic cascade in CRC progression.

### 5.3. Antioxidant and Anti-Inflammatory Effects

The pathogenesis and advancement of colorectal cancer are substantially influenced by two key factors: oxidative stress and persistent inflammation. The former induces genomic instability through DNA damage, while the latter creates an environment conducive to tumor growth [57]. As a therapeutic agent, luteolin demonstrates remarkable efficacy in CRC management through its dual antioxidant and anti-inflammatory mechanisms. Recent research has documented luteolin’s capacity to neutralize reactive oxygen species (ROS), thereby protecting colonic cellular integrity from oxidative damage [58]. The compound’s antioxidant effects are further enhanced by its ability to upregulate key enzymatic defenses, including superoxide dismutase (SOD) and catalase (CAT), which collectively contribute to CRC risk reduction.

**Figure 4:**
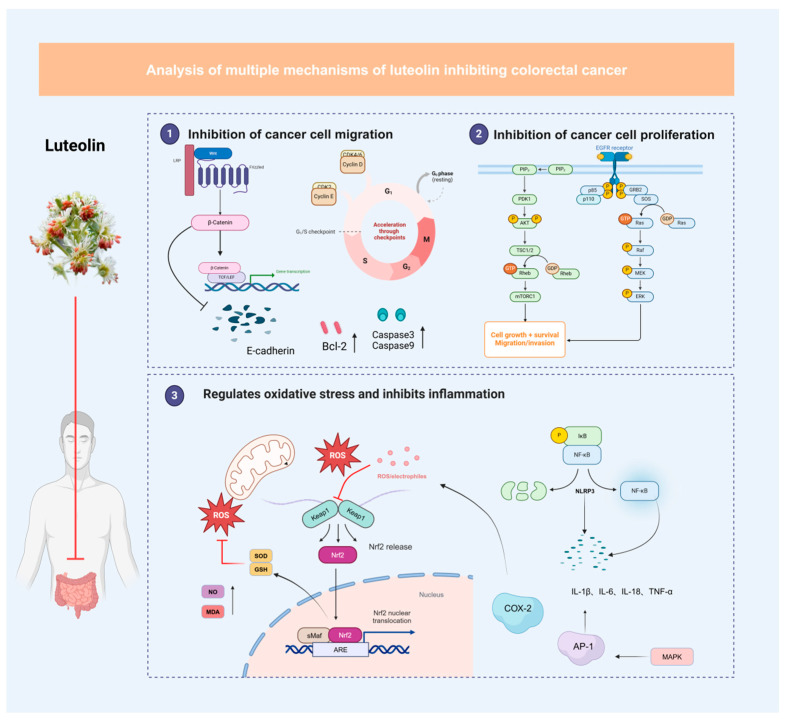
The multi-pathway mechanisms of luteolin in inhibiting colorectal cancer. (The anti-cancer effects of luteolin are achieved through three major pathways: (**1**) Inhibition of cell proliferation and induction of apoptosis—downregulating Cyclin D1/CDK4, activating Caspase-3/9, and modulating the PI3K/Akt pathway; (**2**) suppression of cell migration and invasion—inhibiting MMPs, regulating EMT-related proteins (E-cadherin, N-cadherin, Vimentin), and interfering with the Wnt/β-catenin pathway; (**3**) antioxidant and anti-inflammatory effects—reducing ROS levels, enhancing antioxidant enzyme activities (SOD, CAT), and modulating the NF-κB signaling pathway to create an environment unfavorable for tumor progression).

Furthermore, luteolin exhibits therapeutic potential through its modulatory effect on the NF-kB signaling pathway, thereby attenuating colonic inflammation. Given that persistent intestinal inflammatory conditions significantly elevate CRC risk, luteolin’s anti-inflammatory properties represent a valuable therapeutic approach [51]. The clinical significance of luteolin is particularly evident when administered in combination with conventional anti-cancer therapies, potentially enhancing treatment outcomes [52,59]. This dual protective mechanism—preventing oxidative damage to healthy colonic tissue while simultaneously suppressing inflammation-driven carcinogenesis—underscores luteolin’s therapeutic value (Figure 4).

## 6. Key Considerations

### 6.1. Bioavailability of Luteolin

The bioavailability of pharmaceutical compounds is one of the key factors in assessing their effectiveness as therapeutic agents [60]. Bioavailability refers to the extent to which a drug is absorbed and available in the body, which is often affected by the chemical properties of the drug, the route of administration, and the metabolism and excretion processes in the body [61]. As a flavonoid, luteolin’s water solubility and fat solubility limit its absorption in the body. In vivo studies have shown that luteolin is mainly absorbed through the oral route and enters the blood circulation in small intestinal epithelial cells through passive diffusion or transporter-mediated means [62,63]. Once in the bloodstream, luteolin can distribute to various tissues and organs, especially those with high vascularization and inflammatory activity. The metabolism of luteolin mainly occurs in the liver, and is biotransformed through phase I and phase II metabolic enzymes of the liver. Phase I metabolism usually involves oxidation, reduction, or hydroxylation reactions, while phase II metabolism involves combining preliminary metabolites with sulfuric acid, glucuronic acid, or glutathione to form metabolites that are more soluble in water for easy excretion [64]. Ultimately, the metabolites of luteolin are excreted mainly in urine through the kidneys, and some may also be excreted into the intestines through bile and subsequently excreted in feces. It is worth noting that the bioavailability of luteolin may also be affected by gut microbiota, as gut microbiota can metabolize certain flavonoids and may affect their bioactivity and excretion.

As research progresses, the solubility and bioavailability of luteolin can be significantly enhanced through specific formulation technologies, including microparticle delivery, nanoparticle systems, and solid dispersions [65,66,67]. Recent studies indicate that advanced formulation techniques can markedly improve the water dispersibility and bioavailability of luteolin, thereby augmenting its therapeutic potential for conditions such as inflammatory bowel disease (IBD). For instance, one study demonstrated that the water dispersibility of luteolin was successfully enhanced using a microemulsion system, and its bioavailability was evaluated in a rat model via oral administration. The findings revealed that, compared to untreated luteolin, the area under the concentration–time curve (AUC) of luteolin (WD-L) in the plasma of the microemulsion system increased by 2.2 times, signifying its substantial bioavailability [68]. Furthermore, another study developed a high-loading myofibrillar protein–luteolin complex utilizing green high-pressure homogenization and high-pressure microfluidization technologies. This approach not only optimized the encapsulation efficiency of luteolin within protein-based carriers, but also exhibited favorable release characteristics and antioxidant activity in an in vitro gastrointestinal digestion model.

### 6.2. Analysis of the Potential Toxicity of Luteolin

Luteolin is a widely studied flavonoid known for its potential in treating conditions such as inflammatory bowel disease. However, it is essential to consider its potential toxicity. Research indicates that the toxicity of luteolin may be dose-dependent. At low doses, luteolin typically exhibits favorable biological activity and safety. Conversely, at high doses, in vitro cell experiments suggest that elevated concentrations of luteolin may adversely affect cell viability or induce cell death [69]. Studies in animal models have demonstrated that long-term or high-dose administration of luteolin can lead to weight loss, organ dysfunction, and other health issues. Furthermore, prolonged or repeated exposure to high concentrations of luteolin may impact metabolic organs such as the liver and kidneys. Individual variations, including age, gender, genetic background, and pre-existing health conditions, may also influence an individual’s response to and tolerance of luteolin [70,71].

Thus, future research on luteolin should concentrate on a comprehensive investigation of its toxicity mechanisms, the execution of large-scale clinical trials to assess its safety and efficacy, the examination of individual differences affecting its bioavailability, and the optimization of its structure through the synthesis of new compounds. Additionally, studies should focus on elucidating their mechanisms of action across various disease models and evaluating compatibility with other ingredients to enhance clinical diagnosis and treatment of IBD.

## 7. Discussion

Recent studies have revealed that luteolin exhibits remarkable therapeutic potential in treating inflammatory bowel disease (IBD) and colorectal cancer (CRC). While the pathogenesis of IBD involves multiple complex factors, the disruption of intestinal mucosal barrier function remains a crucial element. Luteolin achieves its therapeutic effects through modulation of immune responses to maintain immunological homeostasis. Its robust anti-inflammatory and antioxidative properties contribute to the reduction in oxidative stress and intestinal inflammation. Moreover, it plays a role in modulating the gut microbiota composition, promoting intestinal health. Ultimately, luteolin contributes to intestinal epithelial restoration and strengthens the mucosal barrier function.

In terms of CRC treatment, luteolin exhibits diverse therapeutic mechanisms. Initially, it effectively suppresses CRC cell proliferation and triggers apoptosis through cell cycle regulation and activation of apoptotic cascades. Subsequently, luteolin impedes CRC cell migration and invasion via MMP downregulation and EMT modulation. Additionally, its potent antioxidative and anti-inflammatory characteristics generate an environment unfavorable for tumor progression, highlighting its significant potential in cancer therapeutics.

The therapeutic potential of luteolin in managing both IBD and CRC has received widespread acknowledgment. To optimize its therapeutic outcomes, advances in formulation technology are particularly significant. Recent developments in formulation strategies, including polymeric micelles and stimuli-responsive nanoparticles, have established new avenues for luteolin application. These technological advances not only enhance luteolin’s solubility and stability but also enable targeted drug delivery while reducing drug wastage and adverse effects. Such formulation innovations provide substantial support for luteolin’s application in IBD and CRC treatment.

However, despite luteolin’s promising potential in treating colorectal cancer and IBD, research gaps exist regarding its role during the adenoma phase in the progression from enteritis to colorectal cancer, and the potential toxicity of luteolin warrants attention. Future research directions should emphasize the comprehensive investigation of luteolin’s mechanisms and biological toxicity in treating intestinal adenomas, further elucidation of its toxicological mechanisms, and implementation of large-scale clinical trials to assess its safety profile and therapeutic efficacy.

## 8. Conclusions

This study provides a comprehensive analysis of luteolin’s therapeutic mechanisms in IBD treatment and its applications in colorectal cancer management. In addressing IBD, luteolin’s effectiveness operates through several key pathways: regulation of immune cell differentiation, attenuation of inflammatory responses, protection against oxidative stress damage, regulation of gut microbiota, and enhancement of intestinal barrier integrity. Regarding colorectal cancer, luteolin demonstrates substantial anti-tumor activity by suppressing cancer cell growth, triggering programmed cell death, inhibiting cellular motility and invasion, while exhibiting significant antioxidative and anti-inflammatory properties. However, various challenges remain in both therapeutic applications. The intricate pathophysiology of IBD and cancer development encompasses numerous signaling molecules and pathways, but existing studies largely examine isolated mechanisms, leading to gaps in understanding the complete therapeutic process. Although substantial evidence from in vitro and animal research supports luteolin’s effectiveness, its clinical efficacy in human subjects requires additional verification through clinical studies. Furthermore, research on luteolin’s intervention during the transitional phase from IBD to colorectal cancer, particularly at the adenoma stage, remains limited. Moving forward, research priorities should focus on examining luteolin’s impact during this critical transition period, uncovering its complete mechanism of action through multiple experimental strategies, and implementing comprehensive clinical trials to assess its safety profile and therapeutic efficacy. Additionally, resolving issues concerning luteolin’s bioavailability and potential toxic effects will be essential for successful clinical implementation. Nonetheless, the remarkable therapeutic potential of luteolin in both IBD and colorectal cancer necessitates ongoing research to enhance its clinical application.
